# Enhancing cranial defect repair in rats: investigating the effect of combining Total Flavonoids from Rhizoma Drynariae with calcium phosphate/collagen scaffolds

**DOI:** 10.1186/s13018-023-04398-w

**Published:** 2023-11-28

**Authors:** Lan Yu, Yiyang Shen, Jun Yang, Xiaoyan Feng, Changlong Zhou, Jun Lin

**Affiliations:** 1 Department of Stomatology, Ningbo Medical Center Lihuili Hospital, Ningbo University, Ningbo, Zhejiang China; 2 Department of Stomatology, The First Affiliated Hospital, College of Medicine, Zhejiang University, Hangzhou, Zhejiang China; 3 Department of Cytopathology, Ningbo Diagnostic Pathology Center, Ningbo, Zhejiang China

**Keywords:** Rhizoma Drynariae, Bone defect, Chinese medicine, Adjunctive therapy

## Abstract

**Objective:**

To investigate the therapeutic efficacy of total flavonoids of Rhizoma Drynariae (TFRD) in conjunction with a calcium phosphate/collagen scaffold for the repair of cranial defects in rats.

**Methods:**

The subjects, rats, were segregated into four groups: Control, TFRD, Scaffold, and TFRD + Scaffold. Cranial critical bone defects, 5 mm in diameter, were artificially induced through precise drilling. Post-surgery, at intervals of 2, 4, and 8 weeks, micro-CT scans were conducted to evaluate the progress of skull repair. Hematoxylin–eosin and Masson staining techniques were applied to discern morphological disparities, and immunohistochemical staining was utilized to ascertain the expression levels of local osteogenic active factors, such as bone morphogenetic protein 2 (BMP-2) and osteocalcin (OCN).

**Results:**

Upon examination at the 8-week mark, cranial defects in the Scaffold and TFRD + Scaffold cohorts manifested significant repair, with the latter group displaying only negligible foramina. Micro-CT examination unveiled relative to its counterparts, and the TFRD + Scaffold groups exhibited marked bone regeneration at the 4- and 8-week intervals. Notably, the TFRD + Scaffold group exhibited substantial bone defect repair compared to the TFRD and Scaffold groups throughout the entire observation period, while histomorphological assessment demonstrated a significantly higher collagen fiber content than the other groups after 2 weeks. Immunohistochemical analysis further substantiated that the TFRD + Scaffold had augmented expression of BMP-2 at 2, 4 weeks and OCN at 2 weeks relative to other groups.

**Conclusions:**

The synergistic application of TFRD and calcium phosphate/collagen scaffold has been shown to enhance bone mineralization, bone plasticity, and bone histomorphology especially during initial osteogenesis phases.

Bone defect, a multifaceted pathology prevalent in medical science, can emanate from a multitude of sources such as trauma, inflammation of bone tissue, tumor excision, or congenital malformations [1, 2]. This condition profoundly affects patients’ quality of life, necessitating comprehensive treatment approaches. Presently, bone grafting, which can be either autogenous or allogeneic (originating from a different individual), stands as the conventional method for addressing bone anomalies [3]. However, both techniques present unique challenges. Allograft transplantation may encounter rejection and obstacles in revascularization, while autogenous bone transplantation may give rise to pain at the donor site and complexities in achieving congruence with the recipient bone structure [4].

Calcium phosphate (CP), as the primary inorganic constituent, along with collagen and water as the principal organic components, constitutes the structural matrix of bone tissue [5]. CP and collagen scaffolds, being the two most prominent components, have been shown exceptional biocompatibility, osteoconductivity, and osteoinductivity for bone critical-size defect repair [6, 7]. Critical-size defect (CSD) is the smallest in diameter bone defect that does not heal spontaneously [8]. The research conducted by Kim et al. substantiates that hydroxyapatite demonstrates notable osteocompatibility, effectively facilitating new bone ingrowth [9]. Furthermore, the application of medical-grade polycaprolactone/tricalcium phosphate (mPCL-TCP) combined with collagen scaffold and rhBMP-2 has been observed to enhance bone quality significantly in the treatment of critical bone defects [10]. Additionally, the study by Chatzipetros E et al. reveals that the incorporation of nano-hydroxyapatite/chitosan scaffolds plays a pivotal role in new bone formation in rat calvarial CSD, as evidenced by histomorphometric analysis and cone beam computed tomography (CBCT) measurements [8, 11].

Nevertheless, when bone defects reach critical-size, patients may be confronted with extended recovery durations, subsequently amplifying the risk of treatment-associated complications such as infection and bone malformation [12, 13]. Future inquiries must concentrate on expediting bone regeneration, curtailing the temporal scope of treatment, and obviating the emergence of complications.

Traditional Chinese Medicine (TCM) offers an expansive repository of therapeutic modalities, amenable to enhancement through integration with contemporary medical doctrines [14]. Such fusion may foster the inception of novel solutions and strategies for recalcitrant clinical conundrums. Additionally, this amalgamation aids in deciphering the underlying mechanisms by which TCM manifests its therapeutic impact, thereby consolidating a scientific framework for its clinical translation [15–17].

Several research endeavors have substantiated the therapeutic potential of the Chinese herb Total Flavonoids of Rhizoma Drynariae (TFRD), a compound routinely utilized in formulations for fracture recuperation and osteoporosis management [18]. Prevailing literature underscores TFRD’s capacity to potentiate osteoblast differentiation and mineralization processes [19]. Furthermore, scholarly investigations have postulated TFRD’s ability to curtail the bone resorption functions of osteoclasts, thereby forestalling bone depletion [20]. In vivo studies have corroborated that TFRD enhances trabecular bone count and bone mineral density (BMD), optimizes bone tissue architecture, and galvanizes novel bone synthesis [20, 21]. Moreover, extracts of TFRD have been shown to catalyze bone proliferation at the peripheries of lesions in New Zealand white rabbit parietal bones [22]. In a synergistic approach, the amalgamation of TFRD and calcium derivatives arrested the diminution of collagen fibers in the femur, thereby bolstering the resurgence of nascent bone or cartilage tissue in ovariectomized rats [23]. While its propensity to promote bone healing is recognized, the precise molecular pathways underlying its action remain nebulous. In the current study, we administered TFRD in conjunction with a calcium phosphate/collagen scaffold to rats with cranial defects, monitoring the dynamic shifts in bone repair and correlated factors to scrutinize the influence of TFRD on the osteogenic differentiation of the calcium phosphate/collagen scaffold and its mechanistic principles. The insights gleaned from this investigation may contribute significantly to the evolving paradigm of “integrative medicine,” a nexus that harmonizes traditional Chinese and Western medical philosophies.

## Materials and methods

### Animals

**Fig. 1:**
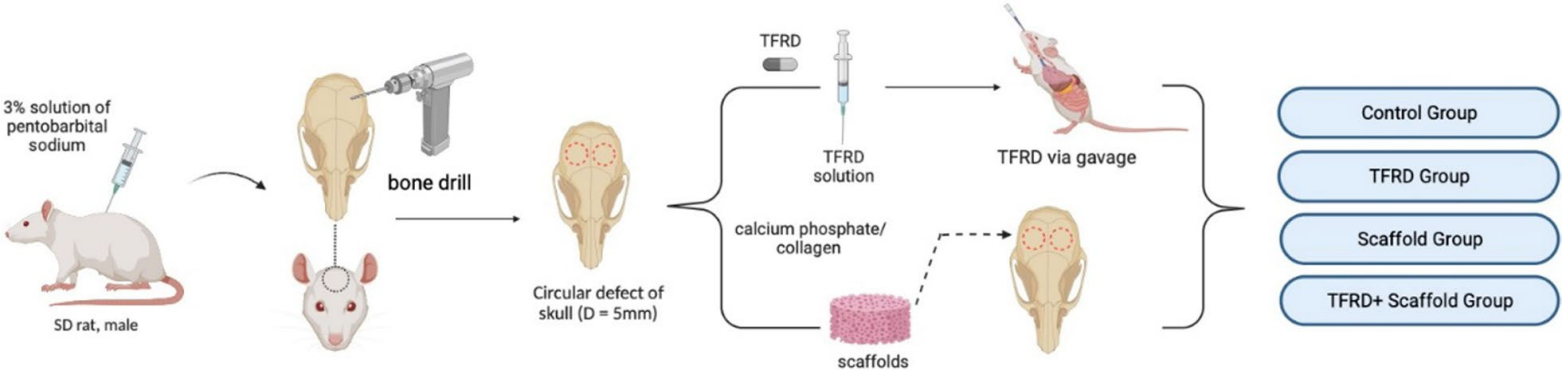
Displayed the schematic representation of the experimental procedure

Male SD rats of SPF grade (aged 10–12 weeks, weighing between 260 and 280 g) were sourced from Hangzhou Ziyuan Experimental Animal Technology Co., Ltd (licence number: SCXK (Zhe) 2019-0004). These subjects were accommodated at the Experimental Animal Centre of the First Hospital of Zhejiang University School of Medicine, three per cage, under meticulously regulated environmental parameters: (25 ± 1) ℃ temperature, (55 ± 5) % relative humidity, and a 12-h photoperiod cycle. The experimental subjects were afforded sterilized water and food ad libitum. The schematic diagram of the experimental process is shown in Fig. 1. The protocol underwent comprehensive review by the Experimental Animal Ethics Committee at the First Affiliated Hospital of Zhejiang University School of Medicine (No. 20221125), ensuring full adherence to the presiding guidelines set forth by the Experimental Animal Ethics Committee.

### Establishment of animal models

**Fig. 2:**
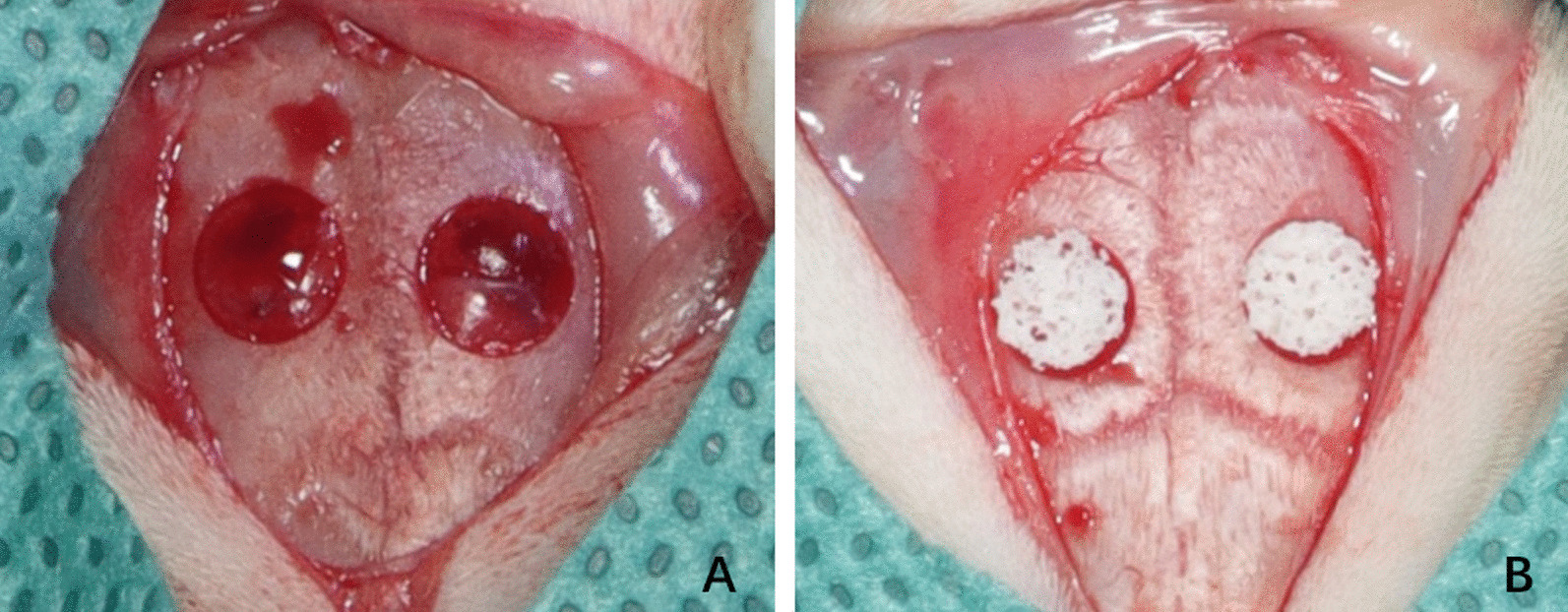
Preparation of cranial defects **A** Cranial defects were modeled in preparation, each defect being 5 mm in diameter and 3–4 mm apart. **B** Placement of calcium phosphate/collagen scaffolds at the site of cranial defects

A period of fasting extending 12 h was mandated for all rats preceding the surgical intervention. The subjects were immobilized in a prone orientation on the surgical apparatus, employing a 3% solution of pentobarbital sodium at a dosage of 1.5 ml per kilogram. Following the removal of the superficial hair layer and implementation of sterile towels, aseptic conditions were rigorously maintained. An aseptic saline solution coupled with sterile gauze was deployed to abolish residual iodine scrub. A subcutaneous administration of 0.5 ml of 1% (wt/vol) lidocaine amalgamated with 1:100,000 epinephrine was performed along the sagittal median of the cranium. A longitudinal incision of 1.5 cm was artfully crafted, extending across the cranial region from the nasal bone to the caudal segment. Two pivotal defects, each 5 mm in diameter, were precisely sculpted utilizing a circular bone drill amid continuous saline irrigation. These defects were strategically spaced 3-4 mm apart, as depicted in Fig. 2. Subsequently, 36 Sprague–Dawley rats were allocated, in a randomized fashion, into four distinct cohorts, namely Control group (*n* = 6), TFRD group (*n* = 6), Scaffold group (*n* = 6), and TFRD + Scaffold group (*n* = 6). Post-implantation of the material germane to each group into the defect regions, the periosteum was scrupulously sutured in a tiered fashion. Postoperatively, the subjects were granted unrestricted access to nourishment, and intramuscular injections of penicillin were administered daily at a dosage of 40,000 U for three successive days. Upon the conclusion of the prescribed time intervals (2, 4, and 8 weeks), the rats within each group were humanely euthanized, allowing for the collection of biological samples for ensuing examination and analysis.

### Treatment

Total Flavonoids of Rhizoma Drynariae (TFRD) constitute a salient component within Qianggu Capsules (fabricated by Beijing Qihuang Pharmaceutical Co., Ltd, batch number: Z20030007, net weight: 0.25 g; each capsule encompasses 0.18 g TFRD). Both TFRD and TFRD + Scaffold cohorts were administered Qianggu Capsules solution, with the equivalent volume sustained for a duration of 8 weeks. The dosage, congruent with 0.22 gkg^−1^d^−1^, was standardized according to the animals’ body surface area, whereas the control group received a parallel volume of 0.9% NaCl. Throughout the intervention period, weekly assessments of the subject body weight were conducted, facilitating the corresponding adjustment of the dosage. Gastric intubations were administered daily (commencing on the immediate postoperative day and persisting until the designated sampling day), culminating in an overall intervention span of 8 weeks.

### Micro-computed tomography (Micro-CT)

**Fig. 3:**
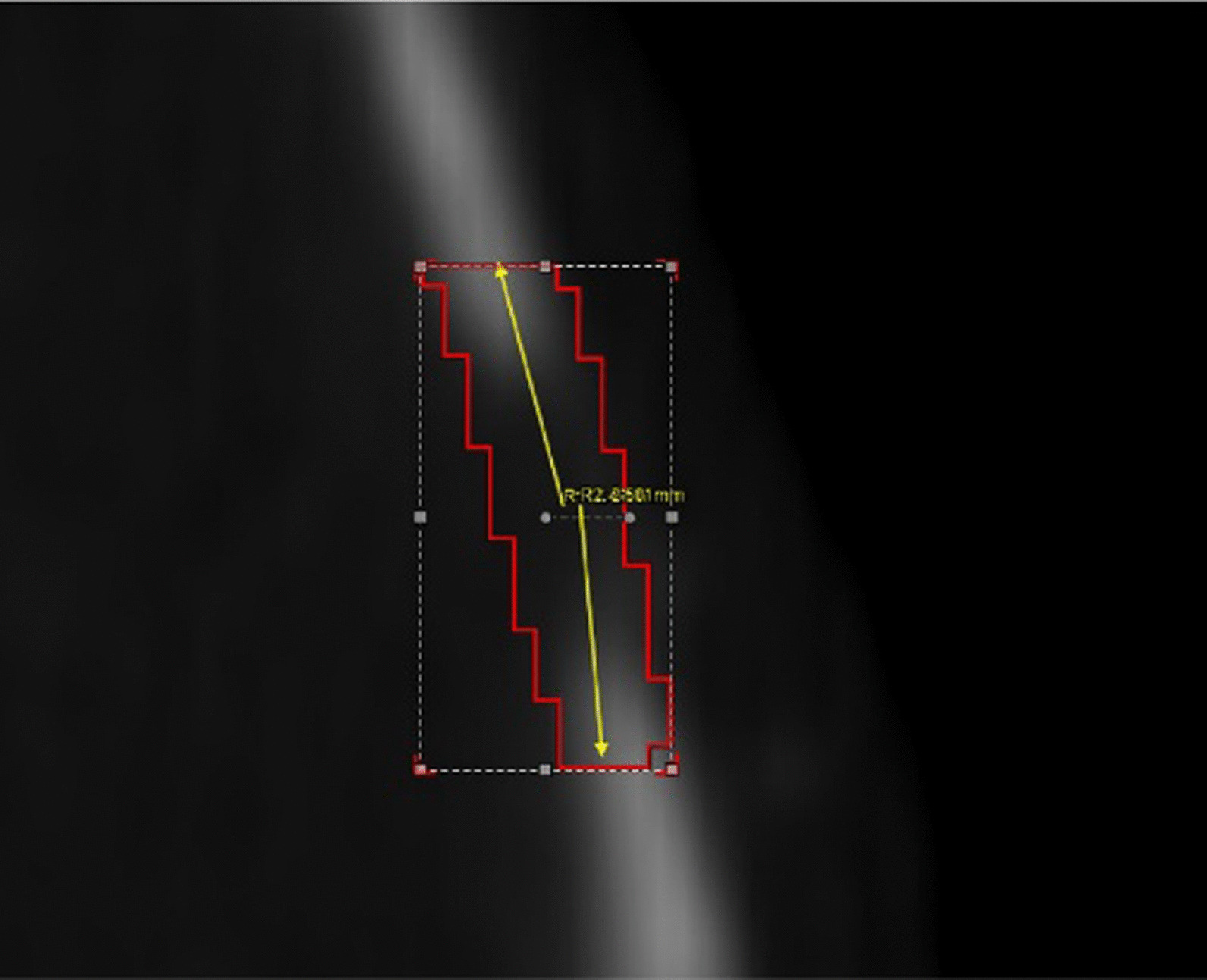
Schematic diagram of the region of interest (ROI) measurement of a bone defect. The system automatically recognizes the sagittal midpoint of cranial defects, yellow arrows indicate 2.5 mm side extensions and the red portion is the ROI area

Antecedent to euthanasia, anesthesia was dispensed to the subjects, positioning them in a supine alignment within the Siemens Inveon integrated micro-PET-CT scanner (Siemens Preclinical Solution USA, Inc., Knoxville, TN, USA). The micro-computed tomography (CT) scanning parameters were set at an 80 kV X-ray tube voltage, 500 uA current, 150 ms exposure duration, and 120 rotational steps. Subsequent image analysis was conducted utilizing the Inveon Research Workplace 4.1 (Siemens, Erlangen, Germany). The bone defect region of interest (ROI) was delineated as the segment extending 2.5 mm to either side from the sagittal midpoint of the predominant skull defect, as shown in Fig. 3. The bone mineral density (BMD), synonymous with bone mineral density, was quantified within this ROI, serving to gauge bone mass and structural integrity.

### Histological analysis

Consequent to the micro-CT examination, the subjects were terminated under profound anesthesia, followed by the extraction and fixation of the cranium in 10% ethylenediaminetetraacetic acid (EDTA) formalin for a 3-week period. This was succeeded by a 12-h aqueous rinse and a serial alcohol dehydration process. The final specimens were encased in paraffin and sectioned horizontally at a 4 μm thickness using a tissue microtome. The application of hematoxylin–eosin (HE) staining facilitated the visualization of osteogenic differentiation and vascularization, while Masson staining was employed to delineate the osteoid fibers within the bone defect. The digitized images were acquired via the digital slide scanning system (KF-PRO-400, Ningbo Jiangfeng Biological Information Technology Co. Ltd.). For Masson staining results, the area of blue collagen in the total area of the bone defect was assessed by histomorphometry.

### Immunohistochemical analysis

The paraffin-embedded bone tissue sections were conditioned at 70 °C for a 4-h period, subsequently deparaffinized, and rehydrated. The antigens were retrieved by pancreatin treatment for 20 min at ambient temperature. The ensuing process involved a 3% H_2_O_2_ application for 5 min to obstruct endogenous peroxidase activity, followed by a washing phase utilizing PBS. Non-specific binding was obstructed with normal goat serum for 10 min. Incubation with rabbit BMP-2 (1:1200, YT0498; ImmunoWay) and OCN (1:200, GB112333, Servicebio) was conducted within a moisture-controlled chamber overnight at 4℃, followed by a 30-min incubation with the horseradish peroxidase (HRP) secondary antibody at room temperature. The subsequent application of 3,3’-diaminobenzidine (DAB) effected color development, with counterstaining performed using hematoxylin. Quantitative assessment of positively stained cells and areas was executed utilizing Image J software (3 random visual fields per section, 3 sections per staining, and 3 rats per time point).

## Result

### Gross observations and micro-CT evaluation

**Fig. 4:**
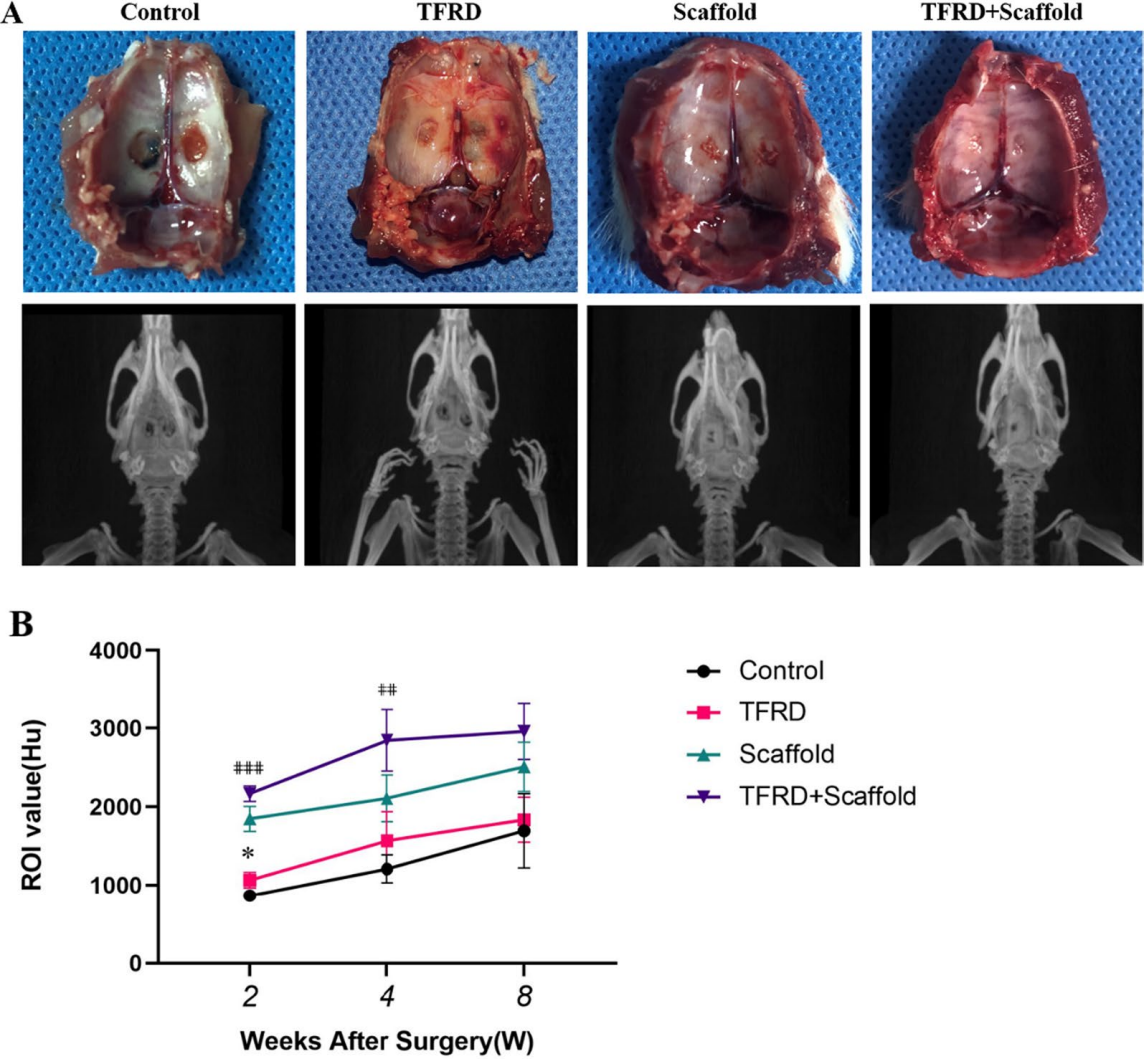
Gross observations and micro-CT evaluation **A** Gross observation and micro-CT examination of representative samples at eight weeks postoperatively. **B** Micro-CT analysis of BMD change over time. *TFRD versus control group. ^⋕^1 TFRD + Scaffold versus Scaffold, (*P < 0.05, ^⋕⋕^*P* < 0.01, ^⋕⋕⋕^*P* < 0.001)

Post-surgery, a comprehensive examination of 36 rats revealed neither accidental mortality nor instances of trauma infection. The subjects maintained an optimal state of health, characterized by stable coat pigmentation, natural posture, unaltered locomotion, consistent dietary intake, and regular bowel function. After an 8-week period, the representative samples showed tactile assessment of the bone defect area in control revealed a soft texture, devoid of bony sensation; microvascular structures remained invisible. Contrarily, the defect in the bone within the Scaffold and TFRD + Scaffold groups had perceptibly diminished, exhibiting a hard, bony texture; especially TFRD + Scaffold group, bone defects substantial recovered, as graphically depicted in Fig. 4A.

Micro-CT quantitative assessments revealed a temporal elevation in BMD across all cohorts, with the TFRD-administered group evidencing an accelerated trajectory. At the bi-weekly checkpoint, the BMD quantification for the TFRD cohort surpassed that of the control group (1064 ± 99.59 versus 865.1 ± 38.82, *p* < 0.05). Intriguingly, the TFRD + Scaffold group’s BMD was substantially elevated compared to the lone Scaffold group (2167 ± 97.45 versus 1847 ± 157.8, *p* < 0.05). By the conclusion of the fourth week, the bone mineral density of the TFRD + Scaffold group perpetuated its dominance over the Scaffold group (2847 ± 391 versus 2107 ± 297.1, *p* < 0.05), with this disparity persisting without any appreciable deviation up to the 8-week mark (Fig. 4B). This connotes a more rapid osseous regeneration within the TFRD + Scaffold cohort relative to its Scaffold counterpart.

The images of the 8-week micro-CT scans were consistent with the naked eye observations. Micro-CT scans demonstrated an absence of marked osteogenesis within the control group’s rat skulls post. A limited proliferation of new bone was detected at the defect’s periphery in the TFRD group. However, in the Scaffold and Scaffold + TFRD groups, the bone defect underwent efficacious repair; particularly in the Scaffold + TFRD group, with the latter particularly evidencing a uniformity in the density of the emergent osseous tissue in juxtaposition with the neighboring cranial structures surrounding the defect. Anchored in these initial findings, our posited hypothesis suggests a symbiotic efficacy of TFRD and scaffold in facilitating osseous recuperation.

### Evaluation of the morphological structure by histological analysis

**Fig. 5:**
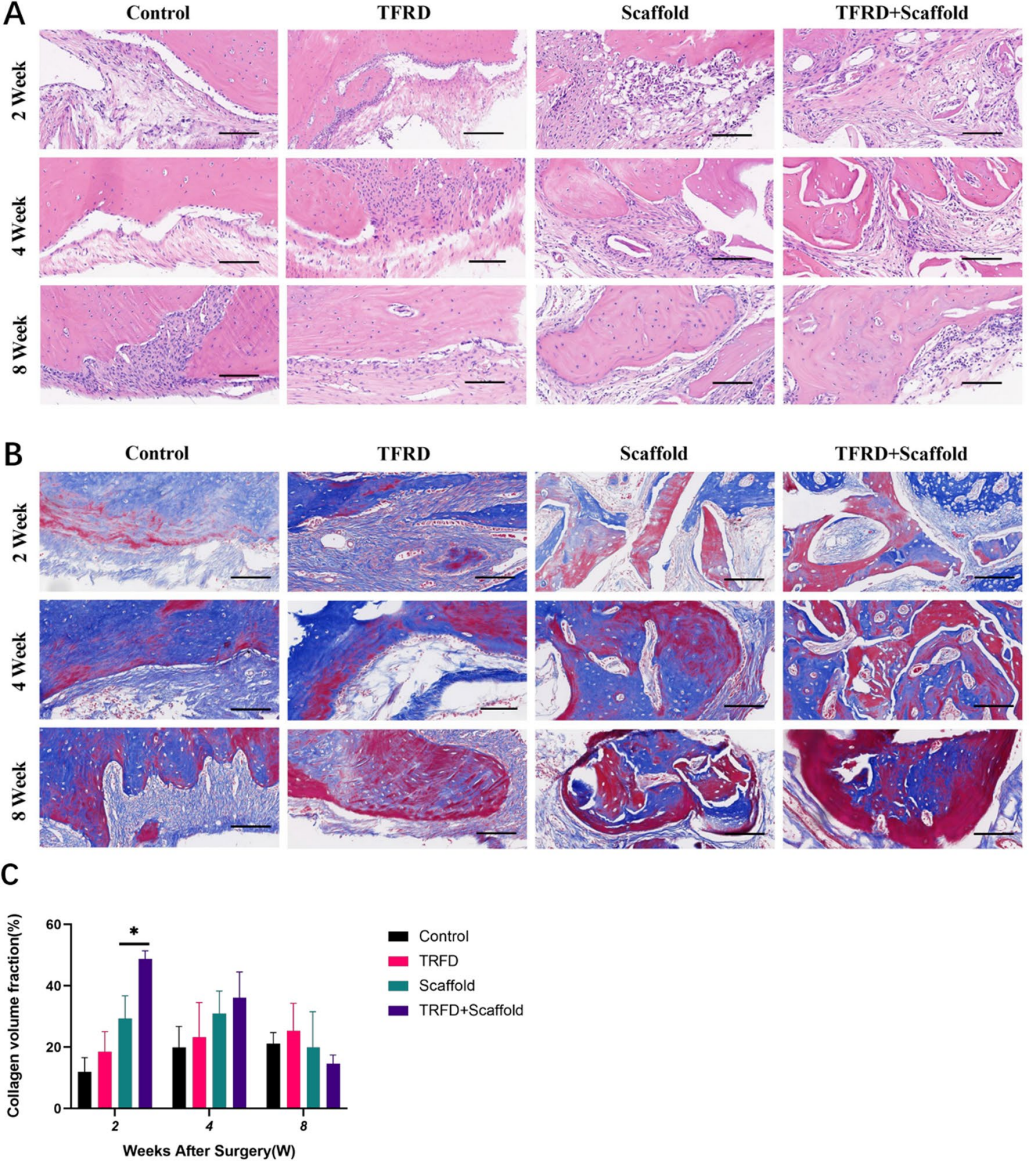
Representative images of HE staining and Masson staining **A** HE staining observed osteogenesis at 2, 4, and 8 weeks in each group. **B** Matson staining assessed the maturity of bone tissue at 2, 4, and 8 weeks in each group. Images were captured at 10× microscopy, with a scale bar shown at 100 μm. **C** Statistical analysis of collagen volume fraction at week 2, 4, 8. TFRD versus control group and TFRD + Scaffold versus Scaffold, (^⁎^*P* < 0.05)

The histological interpretation, facilitated by HE staining, disclosed that the control groups predominantly contained cartilage during the bi-weekly and quad-weekly observations. Within the TFRD contingent, a profusion of active osteoblasts was discernible at the peripheries of the osseous deficits. Conversely, both the Scaffold and the TFRD + Scaffold assemblies were characterized by the presence of extensive regions of neo-osteogenesis, typified by newly formed bone, bone marrow, and osteoid matrix (Fig. 5A). By the culmination of the 8-week period, relative to its counterparts, TFRD + Scaffold group manifested significantly developed mature bone tissues at the defect site.

The maturity of bone tissue was evaluated via Masson staining. Masson staining identified trabecular bone and mature bone tissues in red, collagen fiber and juvenile bone tissues in blue, and the osteoid in pink. Initial results, two weeks post-intervention, depicted conspicuous fibrous and osteogenic regions across all experimental groupings. By the 8-week marker, the TFRD cohort was typified by an expansive osteogenic region suffused with a prominent dark red tone. Meanwhile, Masson staining image analysis showed that TFRD + Scaffold group exhibited a significantly higher mineralized collagen fiber content after two weeks than that of the other groups, twice as much as Scaffold group in comparison (Fig. 5c). Collagen fiber content and neonatal bone tissue decreased, and mature bone tissue increased as time progressed. Consequently, the Masson staining underscored the propitious role of TFRD in augmenting early bone regeneration, and concurrently highlighted that the TFRD + Scaffold conglomerate boasted more advanced bone tissues within the bone defect location.

### Immunohistochemical evaluation

**Fig. 6:**
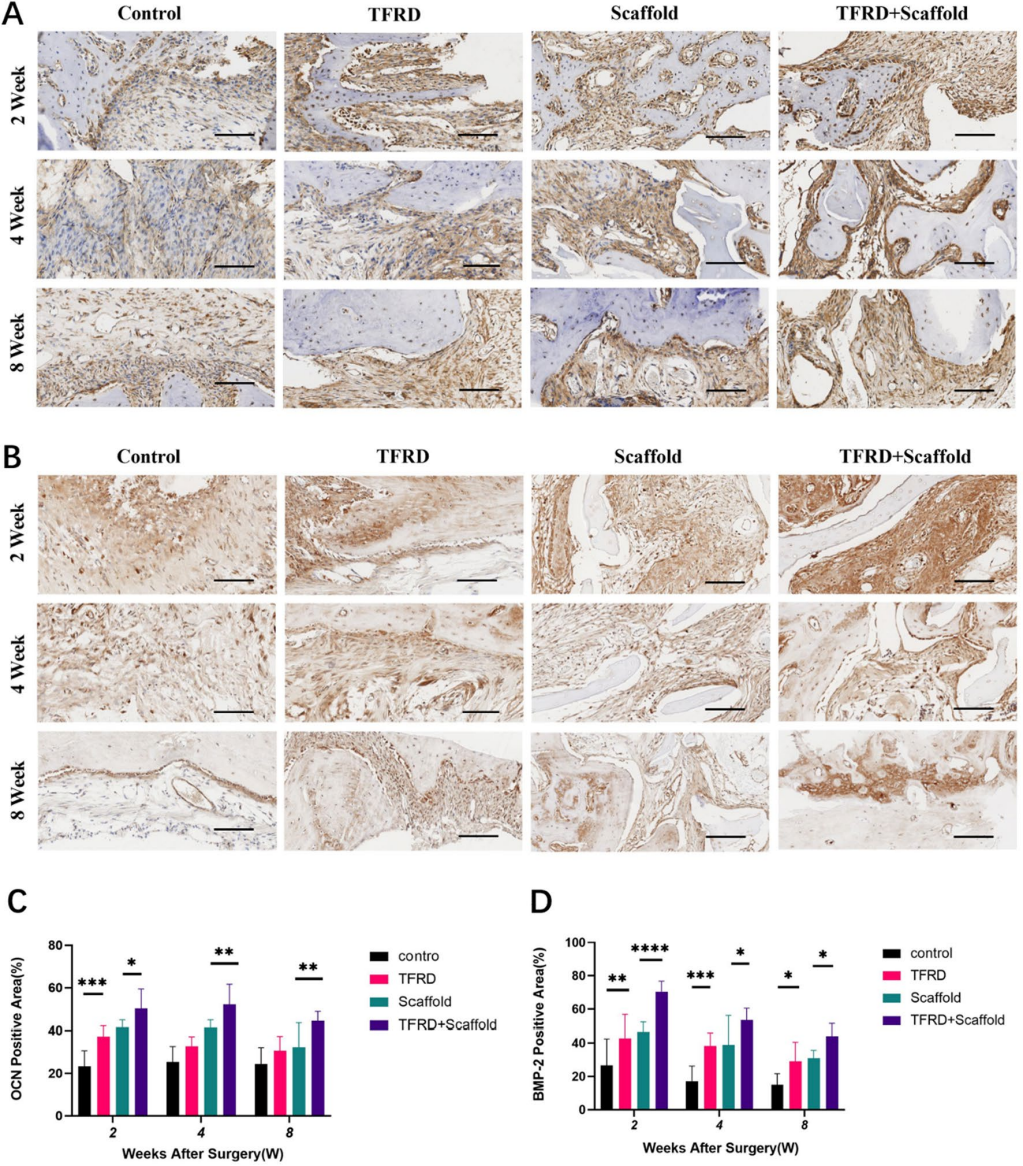
Representative images of IHC staining **A** IHC staining of OCN at the cranial defect perimeters. **B** IHC staining of BMP-2 at the cranial defect perimeters. Images were captured at 10× microscopy, with a scale bar shown at 100 μm. **C** Bar charts show OCN positive area (%) calculated by densitometry of IHC images. **D** Bar charts show BMP-2 positive area (%) calculated by densitometry of IHC images. TFRD versus control group and TFRD + Scaffold versus Scaffold, (*P < 0.05, **P < 0.01, ***P < 0.001, ****P < 0.0001)

The immunohistochemical (IHC) analysis probed the expression of OCN and BMP-2 antibodies at the cranial defect perimeters. OCN-expressing osteoblasts and BMP-2, which is discernible in pluripotent mesenchymal cells, osteoblasts, bone matrices, and associated tissues, both were marked by a brown or yellowish-brown cytoplasmic coloration (Fig. 6A and B). Our results clearly showed that both OCN and BMP-2 have high expression in the TFRD treated groups compared to the control group at the two-week juncture. Furthermore, The TFRD + Scaffold group demonstrated significant elevations in BMP-2 and OCN levels in comparison with the Scaffold alone group from two weeks to eight weeks (Fig. 6C and D). These observations lend credence to the proposition that TFRD catalyzes the release of osteogenesis-associated proteins, partially through the potentiation of the BMP-smad signaling conduit in osteoblasts, thereby fortifying osteogenesis. This further insinuates potential osteogenic synergism attributable to TFRD.

## Discussion

Despite the commendable success rate achieved with calcium phosphate/collagen scaffolds in clinical applications, the quest for expedited bone regeneration and abbreviated treatment durations persist as pivotal frontiers in osteogenesis research [24]. In this context, Traditional Chinese Medicine (TCM) presents a compelling adjunct to surgical interventions. Our present investigation elucidated that the bone healing proficiency of the TFRD cohort markedly surpassed that of the control group. Notably, this enhancement in bone regenerative capability was conspicuously amplified when TFRD was amalgamated with scaffolding substrates, especially during the incipient stages of osteogenesis.

A plethora of empirical endeavors have underscored the salutary influences of both TFRD and calcium phosphate/collagen scaffold in bolstering bone reparative capabilities. To meticulously discern the potential synergistic ramifications of these two entities, we meticulously curated two-by-two comparative matrices within our experimental blueprint: juxtaposing the Control group against the TFRD cohort, and the Scaffold assembly against the TFRD + Scaffold ensemble. Subsequent evaluations spanned imaging diagnostics, histological analyses, and immunohistochemical assessments.

Guo Ying et al. elucidated that TFRD facilitates the osteogenic differentiation of rat bone marrow stem cells (BMSCs), amplifying the expression of pivotal factors such as β-catenin, lymphoid enhancer factor I (LEF-l), and cyclin D mRNA during the nascent phases of differentiation. Intriguingly, TFRD attenuates the expression of these factors as differentiation matures. This observation underscores the hypothesis that TFRD’s primary contribution to osteoblast differentiation predominantly occurs during the early differentiation phases [25]. Jiang Ziwei et al. delved into the temporal effects of TFRD on osteogenesis efficacy, utilizing tibia distraction osteogenesis in rats as a model system. The empirical findings elucidated that the group treated with the flavonoids throughout the entire experimental period exhibited notably superior results in terms of X-ray scores and bone mineral density when juxtaposed with groups receiving interventions during the distraction phase alone or both the distraction and mineralization phases. In summation, an early and consistent administration of TFRD can significantly augment the quality of bone formation in the distraction osteogenic region of rats [26]. Our results are in line with the literatures. The micro-CT BMD analysis showed that the TFRD + Scaffold assembly showcased a superior trajectory and velocity of bone density amplification in contrast to the sole scaffold group during the initial two and four-week periods, which demonstrated TFRD may have favorable consequences concerning precocious osteogenesis.

Collagen fibers within the bone tissue matrix are crucial for osteoblast maturation and matrix mineralization. Type I collagen, a primary component of the bone matrix, facilitates the expression of osteoadhesins, osteocalcin, and bridging proteins, thereby enhancing matrix mineralization during the mineralization phase [27]. In vivo studies at the 2-week mark revealed that the collagen content in the TFRD + Scaffold group was significantly higher than that in the Scaffold group alone. However, there was a gradual decrease in collagen content in the TFRD + Scaffold group at weeks 4 and 8. These observations indicate that TFRD integrated with calcium phosphate/collagen scaffolds may induce early osteogenesis and expedite matrix mineralization.

The principal exhibition of TCM’s facilitation in osteogenic repair can be elucidated in the following dimensions: Initially, promoting blood circulation to dredge collaterals, thereby the prevention and elimination of blood clots mitigate aseptic inflammation and trauma to soft tissues [28]. Subsequently, the enhancement of blood circulation, vascular wall permeability, ion exchange, local deposition of trace elements such as zinc, iron, copper, manganese, and calcium salts occurs [29]. Further, there is stimulation of local collagen synthesis at the fracture terminus within the bone matrix, and the secretion and synthesis of bone growth factors such as BMPs, VEGF and OCN.

The therapeutic efficacy of TFRD in fracture recuperation and osteoporosis management is elucidated through its intricate modulation of two distinct molecular signaling pathways. Firstly, TFRD facilitates the activation of osteogenesis-associated signaling cascades within bone marrow stem cells (BMSCs) and osteoblasts [30]. Notably, pathways such as Wnt/β-catenin, MAPK, and Smad are intricately linked to bone synthesis [31]. Conversely, TFRD mitigates osteoporosis by impeding bone resorption and attenuating bone loss. The critical pathways orchestrating this inhibitory action on bone resorption encompass CTSK and OPG/RANKL/RANK [18, 32]. It is noteworthy that while the majority of therapeutic agents typically target either bone resorption or synthesis singularly, TFRD exhibits a dual modulatory influence on both these processes [33, 34].

As an integral component of the BMPs family, BMP-2 assumes an osteogenic mantle by orchestrating the transcription of genes germane to osteogenesis and by catalyzing the Smads protein signaling mechanism. [35] BMP-2 exerts considerable osteoinductive activity, signifying its capability to directly increase cell augment cellular calcification and instigate new bone formation in conjunction with osteoblast maturation [15, 20]. Data from our investigation delineate that the expression of BMP-2 across all experimental cohorts reached zenithal levels two weeks post-gavage, subsequently waning thereafter. However, consistently across all evaluated intervals, the TFRD + Scaffold group exhibited superior BMP-2 expression compared to the Scaffold group, and the TFRD group surpassed the Control group. These findings are congruent with extant literature, illustrating that TFRD elevates BMP-2 expression, thus favoring bone production and differentiation.

Osteoblasts proceed through four sequential stages during bone formation: proliferation, extracellular matrix maturation, extracellular matrix mineralization, and apoptosis. [36] Accordingly, the investigative approach to augment the pace of induced bone healing encompasses the stimulation of osteoblast proliferation, differentiation, mineralization, and the release of augmented osteogenic-related proteins. Osteocalcin (OCN), a ubiquitous non-collagenous protein in bone tissue synthesized by osteoblasts and marked by a swift turnover rate, functions as a precise and unerring indicator of osteoblast activity [37]. The results manifested that the group receiving scaffold combined with TFRD gavage displayed increased bone growth and mineralization in both the central and peripheral regions of the bone defect area, culminating in the upregulation of osteogenic-associated factors such as OCN. The escalated OCN positive expression in the TFRD-Scaffold group surpassed that achieved by scaffold alone, corroborating the enhancement of osteoblasts’ bone formation functionality and the remodeling process facilitated by TFRD.

Through rigorous animal-based experimentation, our study culminates in the conclusive assertion that the confluence of TFRD and calcium phosphate/collagen scaffolds exudes a distinct synergistic efficacy. TFRD amplifies bone repair capabilities, primarily by galvanizing the expression of osteogenesis-associated proteins like BMP-2 and OCN, especially during the nascent stages of bone regeneration. This intricate tapestry of actions provides clarity on the synergistic potential when TFRD is integrated with calcium phosphate/collagen scaffolds. Such findings bolster the proposition for TFRD’s incorporation as a potent herbal therapeutic for addressing bone defects.

## Data Availability

The initial data used to support the findings of this study are available from the corresponding author upon request.

## Abbreviations

TFRD: Total Flavonoids of Rhizoma Drynariae
BMP-2: Bone morphogenetic protein 2
OCN: Osteocalcin
CP: Calcium phosphate
